# Vitamins and Human Health: Systematic Reviews and Original Research

**DOI:** 10.3390/nu15132888

**Published:** 2023-06-26

**Authors:** Tyler Barker

**Affiliations:** 1 Sports Medicine Research Institute, The Ohio State University Wexner Medical Center, Columbus, OH 43202, USA; tyler.barker@osumc.edu; 2 Department of Orthopaedics, University of Utah, Salt Lake City, UT 84108, USA; 3 Nutrition and Integrative Physiology, University of Utah, Salt Lake City, UT 84112, USA

Vitamins are a group of organic compounds essential to physiological functions in the body. This Special Issue features systematic review and original research articles of vitamins in health and disease. Among other topics, the association of endogenous vitamin levels with disease risk, the therapeutic role of vitamin supplementation in various diseases, and an analytical method for measuring vitamin D and K content in dietary supplements are discussed. Below is a summary of the articles included in this Special Issue.

Vitamins are involved in cellular processes and are associated with the development or prevention of malignant diseases. To summarize the role of select vitamins (i.e., A, B-complex, C, D, E, and K) in oncogenesis, Venturelli and colleagues [1] reviewed the association between micronutrient concentrations in the blood or tissues and cancer risk and, importantly, examined if vitamin concentrations potentially serve as biomarkers for early cancer detection. Despite heterogeneity in the results, a low endogenous concentration of select vitamins was associated with an increased risk of some cancers. Along with the likelihood of DNA insult due to oxidative stress or structural fragility and epigenetic aberration, Venturelli and colleagues [1] recommended that longitudinal tracking of vitamin concentrations in the body might provide diagnostic and prognostic value as opposed to assessments at a single time point. Additional research assessing vitamin levels as biomarkers in cancer and cancer-related conditions (e.g., cachexia) is warranted.

Dementia is a progressive cognitive impairment syndrome that interferes with activities of daily and independent living. This Special Issue contains an extensive systematic review of vitamin supplementation and dementia [2]. The review by Martinez and colleagues [2] found folic acid and thiamine supplementation alone or in combination improved cognitive performance, while conflicting results were reported with combined folic acid and vitamin B12 supplementation. Cognitive performance appeared to respond favorably to ascorbic acid (vitamin C) and high-dose vitamin E supplementation when taken separately, but more research is necessary to support the use of vitamin E due to a limited number of studies investigating its role in dementia. Cognitive performance dramatically varied in response to vitamin D supplementation, and thus, the results were inconclusive regarding the potential benefit of supplemental vitamin D on cognition [2]. Delineating the association of and identifying the mechanisms governed by diverse vitamins with cognitive impairment could reveal supplemental interventions that complement existing standard of care models in this challenging condition.

Vitamin A refers to a group of fat-soluble retinoids, and vitamin A deficiency is associated with increased susceptibility to infections. A thorough systematic review by Sinopoli and colleagues [3] summarized the role of supplemental vitamin A to protect against viral infections. The heterogenous findings were across and within viral families. Overall, vitamin A supplementation was relatively safe, but no meaningful results were found regarding its ability to prevent viral infections. Encouraging results were, however, described for the management of some viral diseases with vitamin A supplementation.

The B-complex vitamins are important early on and during the different stages of the life cycle. A comprehensive assessment of the different B vitamin requirements is reviewed across the life cycle by Ali and colleagues [4]. Parallel to the review by Ali and colleagues [4], another systematic review assessed the total all-source intake of folate in women of childbearing age and during pregnancy in high-income countries with food fortification programs [5]. Ledowsky and colleagues [5] found that women of childbearing age do not receive sufficient folate intake from food alone and almost all women taking a folic-acid-based supplement exceeded the tolerable upper limit of folic acid intake. Based on those findings, the authors [5] concluded that folic acid supplements and the upper tolerable limit require careful review when considering the adverse effects of exceedingly high intakes of folic acid and its subsequent impact on women’s health during childbearing years.

Vargas-Uricoechea and colleagues [6] investigated vitamin B12 status in southwestern Colombia. From their analysis, Vargas-Uricoechea and colleagues [6] found that the prevalence of vitamin B12 deficiency increased with age and in those with type 2 diabetes mellitus. Furthermore, metformin use (i.e., >1 gm/d) increased the prevalence of vitamin B12 deficiency in those suffering from type 2 diabetes mellitus. As the authors suggested [6], these findings may not be consistent in other populations or in other populations with diverse health conditions.

Vitamin C is a water-soluble antioxidant that enhances phagocytic cell function. Endogenous vitamin C levels are often low in patients requiring an autologous hematopoietic stem cell transplant. In a randomized, blinded, placebo-controlled study [7], supplemental (intravenous) vitamin C did not improve neutrophil function, hospitalization, or survival in patients requiring autologous stem cell transplantation for myeloma or lymphoma despite possible lower bacteremia rates. Based on the data from the study, van Gorkom and colleagues [7] concluded that vitamin C supplementation is not advised in myeloma or lymphoma patients.

Excess iodine intake increases reactive oxygen species production and decreases the activity of antioxidant enzymes. In their study, Sun and colleagues [8] investigated the protective influence of vitamin C obtained through fruit, vegetables, and other dietary sources against oxidative damage induced by long-term and excess iodine exposure in experimental rats. In this randomized study, a high dose of vitamin C protected against and a low dose of vitamin C exacerbated oxidative damage.

Low vitamin D (i.e., serum 25-hydroxyvitamin D (25(OH)D)) is routinely associated with poor health and disease. Using Canadian Health Measures data, Yousef and colleagues [9] examined immigrant participants’ health status in relation to chronic disease and vitamin D status. In general, the immigrant participants were healthier than their non-immigrant counterparts for most health status outcomes. The prevalence of chronic disease was higher for those who migrated to Canada as adults (i.e., >18 years of age). In addition, serum 25(OH)D concentrations were lower among the immigrant participants, and, surprisingly, higher in patients with chronic disease. Serum 25(OH)D concentrations were not associated with increased prevalence of chronic diseases but were inversely correlated with chronic-disease-related biomarkers (i.e., blood hemoglobin, total cholesterol/high density lipoprotein ratio, immunoglobulin E, and serum ferritin). These findings indicate that low serum 25(OH)D concentrations are associated with chronic-disease-related biomarkers but not chronic diseases. A multitude of factors regulate endogenous vitamin D levels and additional research is needed to define the serum 25(OH)D relationship with health status deterioration and chronic disease.

Vitamin D deficiency is common among multiple sclerosis patients, and Glabska and colleagues [10] reviewed the role of supplemental vitamin D in mental health in patients with multiple sclerosis. From their review, Glabska and colleagues [10] reported that supplemental vitamin D appeared to have a positive influence on the mental health and quality of life of patients with multiple sclerosis in the majority or all the studies included. However, depression or depressive symptoms were not impacted by supplemental vitamin D in patients with multiple sclerosis.

Zhu and colleagues [11] examined the distribution and determinants of vitamin D binding protein (VDBP) and total, bioavailable (and complementary “non-bioavailable”), and free 25(OH)D in older adults (50–75 years of age). The analysis included genetic, lifestyle, and dietary factors linked to health conditions. The study data indicated that VDBP levels are inversely associated with age and BMI and that they are positively associated with cholesterol and CRP levels [11]. In addition to several correlations, the vitamin D biomarker concentrations (i.e., VDBP and free, total, bioavailable, and non-bioavailable 25(OH)D) differed by VDBP genotype.

Covering a broad spectrum of different autoimmune diseases, Amon and colleagues [12] reported that a high dose of oral vitamin D3 (up to 1000 IU/kg of body mass with a mean daily dose of 35,000 IU) for more than 3.5 years was safe in terms of calcium metabolism and renal function. The protocol consisted of strict dietary and fluid recommendations, and more than 6100 relevant laboratory parameters were monitored. The provocative study by Amon and colleagues [12] provides a substantial amount of data pertaining to the safety of high-dose vitamin D3 in autoimmune patients when monitored under the direct supervision of experienced physicians with expertise in vitamin D and vitamin D metabolism.

Multiple myeloma is a non-common plasma cell malignancy. One of the underlying mechanisms contributing to the etiology of multiple myeloma is immune system dysregulation. Kulig and colleagues [13] reviewed the in vitro and in vivo potential of vitamin D as an adjuvant therapy for multiple myeloma and its application to improve patient outcomes. From the review, Kulig and colleagues [13] summarized compelling evidence from predominantly in vitro studies suggesting that vitamin D could be clinically relevant in managing multiple myeloma as an adjuvant therapy, particularly in combination with chemotherapeutic agents. Rightfully so, however, the review identifies a shortage of clinical trials investigating the application of vitamin D in multiple myeloma.

The adjuvant role of vitamin D was proposed in another review article included in this Special Issue. Renke and colleagues [14] reviewed the association between vitamin D and oxidative stress and cardiovascular disease risk. From their literature search, they [14] reported that vitamin D decreases free radical production, which could have favorable outcomes in treating cardiovascular diseases mediated in part by oxidative stress. Determining the appropriate dose and duration of vitamin D to combat oxidative stress is an intriguing area of research, especially since correcting low vitamin D levels could reduce endothelial damage and cardiovascular disease by decreasing oxidative stress and other mechanisms propagating disease development and progression.

In a review of randomized controlled trials, Guzek and colleagues [15] assessed the impact of supplemental vitamin D on depressive disorders, as well as bipolar depression and postpartum depression. While a few studies have reported the positive influence of supplemental vitamin D with a medium risk of bias, confirmatory evidence identifying the clinical utility of treating depressive disorders or general depression with supplemental vitamin D is lacking.

Niedermair and colleagues [16] reviewed the ability of vitamin D food fortification to achieve a similar 25(OH)D concentration to supplemental vitamin D and at doses found to reduce cancer mortality. Food fortification increased the serum 25(OH)D concentration comparable to equivalent doses of supplemental vitamin D found to lower cancer mortality. The cost of food fortification with vitamin D was lower compared to previously estimated costs of vitamin D supplementation in an older population. Future studies comparing vitamin D food fortification to supplementation are needed to extend these findings.

The article by Starek and colleagues [17] describes a method for analyzing vitamin D (D2 and D3) in conjunction with vitamin K in dietary supplements available on the Polish market. The methodology was sound and cost effective. In the analysis [17], the amount of vitamin D3 in the products analyzed closely resembled the content reported by the manufacturer while the vitamin K2 measured compared to the reported content was more variable.

Aluminum is a ubiquitous and widely used metal that combines with other elements to form different compounds. Renke and colleagues [18] reviewed the deleterious impact of aluminum on human health. The study’s data indicated that aluminum alters clinical and metabolic outcomes. Neurotoxicity is a critical adverse event of aluminum, and aluminum accumulation increases inflammation and oxidative stress. Thus, exposure to aluminum should be kept at a minimum, and additional studies investigating the impact of aluminum on human health are of interest.

In summary, this Special Issue contains articles related to a variety of vitamins in health and disease. Respecting the diverse physiological roles of vitamins and the underlying determinants of endogenous vitamin levels will be important therapeutic and prognostic considerations of future research.
